# *Retracted:* In vivo mecillinam resistance in *Escherichia coli* after pivmecillinam treatment of a urinary tract infection

**DOI:** 10.1002/mbo3.770

**Published:** 2019-01-06

## Abstract

Retraction: Nielsen, KL, Hansen, KH, Knudsen, JD, Frimodt‐Møller, N, Hertz, FB, Jansåker, F., In vivo mecillinam resistance in Escherichia coli after pivmecillinam treatment of a urinary tract infection, MicrobiologyOpen, 2019; e770 (https://doi.org/10.1002/mbo3.770). The above article, published online on 6 January 2019 in Wiley Online Library (wileyonlinelibrary.com), has been retracted by agreement between the authors and John Wiley & Sons Ltd. The retraction has been agreed due to the authors’ self‐reported errors in their data rendering the article’s conclusions incorrect.

Retraction: Nielsen, KL, Hansen, KH, Knudsen, JD, Frimodt‐Møller, N, Hertz, FB, Jansåker, F., In vivo mecillinam resistance in Escherichia coli after pivmecillinam treatment of a urinary tract infection, MicrobiologyOpen, 2019; e770 (https://doi.org/10.1002/mbo3.770). The above article, published online on 6 January 2019 in Wiley Online Library (wileyonlinelibrary.com), has been retracted by agreement between the authors and John Wiley & Sons Ltd. The retraction has been agreed due to the authors’ self‐reported errors in their data rendering the article’s conclusions incorrect.

